# Study of Non-Isothermal Crystallization of Polydioxanone and Analysis of Morphological Changes Occurring during Heating and Cooling Processes

**DOI:** 10.3390/polym8100351

**Published:** 2016-09-28

**Authors:** Yolanda Márquez, Lourdes Franco, Pau Turon, Juan Carlos Martínez, Jordi Puiggalí

**Affiliations:** 1 Chemical Engineering Department, Escuela d’Enginyeria Barcelona Est (EEBE), c/ Eduard Maristany 19, Barcelona 08930, Spain; ymlyolanda@gmail.com (Y.M.); lourdes.franco@upc.edu (L.F.); 2 B. Braun Surgical S.A., Carretera de Terrassa 121, Barcelona 08191, Spain; pau.turon@bbraun.com; 3 ALBA Synchrotron Light Facility, Ctra BP 1413 km 3.3, Cerdanyola del Vallès, Barcelona 08290, Spain; guilmar@cells.es

**Keywords:** polydioxanone, surgical suture, non-isothermal crystallization, lamellar stacking, synchrotron radiation

## Abstract

Non-isothermal crystallization kinetics of polydioxanone (PDO), a polymer with well-established applications as bioabsorbable monofilar suture, was investigated by Avrami, Mo, and isoconversional methodologies. Results showed Avrami exponents appearing in a relatively narrow range (i.e., between 3.76 and 2.77), which suggested a three-dimensional spherulitic growth and instantaneous nucleation at high cooling rates. The nucleation mechanism changed to sporadic at low rates, with both crystallization processes being detected in the differential scanning calorimetry (DSC) cooling traces. Formation of crystals was hindered as the material crystallized because of a decrease in the motion of molecular chains. Two secondary nucleation constants were derived from calorimetric data by applying the methodology proposed by Vyazovkin and Sbirrazzuoli through the estimation of effective activation energies. In fact, typical non-isothermal crystallization analysis based on the determination of crystal growth by optical microscopy allowed secondary nucleation constants of 3.07 × 10^5^ K^2^ and 1.42 × 10^5^ K^2^ to be estimated. Microstructure of sutures was characterized by a stacking of lamellae perpendicularly oriented to the fiber axis and the presence of interlamellar and interfibrillar amorphous regions. The latter became enhanced during heating treatments due to loss of partial chain orientation and decrease of electronic density. Degradation under various pH media revealed different macroscopic morphologies and even a distinct evolution of lamellar microstructure during subsequent heating treatments.

## 1. Introduction

Poly(*p*-dioxanone) (PDX or PDO) is a synthetic poly(ester-ether) with wide applications in the biomedical field due to its excellent properties (e.g., biodegradability, biocompatibility, bioabsorbability, softness, and flexibility) [1]. In relation to polyglycolide, i.e., the first polyester employed for biomedical applications, the chemical repeat unit of PDO has an ether bond and additional methylene groups that provide greater flexibility. In this way, PDO can be used as a monofilar surgical suture, in contrast with the braided polyglycolide suture. PDO can be completely reabsorbed in a period close to six months, with no significant foreign body reaction being observed in the tissues surrounding the implant.

Several companies have commercialized PDO under different trademarks (e.g., PDS II and MonoPlus™ by Ethicon and B. Braun Surgical S.A., respectively) as a long term surgical suture that appears as an ideal wound support for healing periods longer than four weeks. Other biomedical applications such as bone or tissue fixation devices, fasteners, and drug delivery systems [2] should also be considered since the polymer can be easily injection-molded.

Isothermal crystallization of PDO has been extensively studied by different techniques, including polarized optical microscopy and differential scanning calorimetry (DSC) [3,4,5,6,7,8]. Changes in morphological parameters have also been evaluated by small-angle X-ray scattering [9]. Several works have focused on the influence of degradation on morphology and isothermal crystallization behavior [10,11,12].

Surprisingly, only a few reports concern the crystalline structure of PDO and lead to some conflicting results. Thus, early studies pointed to an orthorhombic unit cell containing only two molecular segments with a tight pitch (i.e., the chain axis repeat was shortened by 52% compared to the expected value for an extended zig-zag conformation) [13]. Subsequently, an orthorhombic unit cell with space group *P*2_1_2_1_2_1_ and parameters *a* = 0.970 nm, *b* = 0.751 nm and *c* (chain axis) = 0.650 nm was postulated using X-ray and electron diffraction data [14]. The structural model was also supported by quantum mechanical calculations that indicated a chain periodicity given by a single residue with a TGT(-G)TT conformation and a unit cell containing four molecular segments. Crystals obtained from solution had a variable morphology (i.e., the acute apex angle of lozenge crystals varied with the crystallization conditions) that seems a consequence of the peculiar structure of PDO, where different folds for adjacent chain re-entry exist [14]. 

It is well established that isothermal crystallization of PDO from the melt renders spherulites with a distinctive morphology depending on the crystallization temperature. Specifically, spherulites obtained at high temperature were double-ringed [3] whereas at low temperature they showed a negative birefringence and the typical Maltese cross. Isothermal crystallization kinetic studies linked the different morphologies to two crystallization regimes [3]. Ringed and negative birefringent spherulites were observed by evaporation of concentrated formic acid solutions. It was demonstrated that the *a* crystallographic axis of constitutive lamellae was oriented along the spherulite radius [14].

The knowledge of non-isothermal crystallization kinetics is essential to achieve the proper microstructure and the required properties of a material. The control of crystallization may reduce undesired effects during processing such as excessive anisotropy of shrinkage, warpage, and insufficient dimensional stability [15]. Nevertheless, it should be considered that non-isothermal crystallizations studies are usually limited to idealized situations, in which external conditions such as the cooling rate is kept constant, although in real situations polymers are cooled down at different rates and with high thermal gradients [16]. 

Non-isothermal crystallization of PDO has been scarcely studied despite the fact that melt-processed samples (e.g., bioabsorbable surgical sutures) are obtained under non-isothermal conditions. However, some non-isothermal melt crystallization analyses have been performed from DSC data and by applying Ozawa [17] and Cazé [18] methodologies to evaluate Avrami exponents [4]. Values calculated by Ozawa were dependent on the temperature, whereas two different values of the Avrami exponent were determined depending on the cooling rate [6]. Non-isothermal cold crystallization has also been evaluated by calorimetric analysis [19] and Avrami [20,21], Ozawa [17], and Tobin [22] methodologies. In this case, results suggested that the Avrami method was more effective in describing non-isothermal cold crystallization kinetics, with reported values of Avrami exponent in the 4.5–5.3 range [19].

PDO has a limited use, for example, as blow processed films due to its low crystallization rate and melt strength, together with its high cost and relatively low thermal stability. Therefore, non-isothermal crystallization studies have mainly focused on blends of PDO with other polymers such as poly(vinyl alcohol) [23] and polylactide [24]. It was assumed that the crystallization rate should be enhanced in blends. Thus, an improvement of properties and easier processing were determined. 

The purpose of the present work was to perform a complete analysis of non-isothermal crystallization considering both the overall kinetic process from DSC data and crystal growth rates from optical microscopy data. Furthermore, morphological changes occurring during heating and cooling processes were analyzed by real time synchrotron experiments. Dynamic diffraction data from samples degraded under different pHs were useful in confirming a lamellar organization inside fibers. In this way, recent studies on degraded monofilament sutures constituted by polyglycolide hard blocks highlighted a structural organization with interlamellar and interfibrillar domains [25].

## 2. Experimental Section

Materials: Granulated PDO and processed PDO sutures (Monoplus™, USP 0) were kindly supplied by B. Braun Surgical, S.A (Rubí, Spain). In order to get PDO sutures, granulated polymer was gradually melted along the extruder barrel under an inert atmosphere applying a temperature gradient from 140 to 190 °C. The poly(*p*-dioxanone) polymer melt was metered by a spin pump with a specific throughput and extruded through a spinneret, which shapes the material to a monofilament fiber with a circular cross section. After passing through the spinneret, the fiber was quenched in a cold water bath to stabilize it. In order to obtain good tensile properties, the fiber was drawn and relaxed at certain drawing ratios, passing it through convection ovens. Finally, the fiber was taken up on drums winders. Number and weight average molecular weights determined by GPC were 112,800 and 250,000 for the granulated material and 103,000 and 226,300 for the commercial threads, respectively. 

Measurements: Molecular weights were estimated by size exclusion chromatography (GPC) using a liquid chromatograph (Shimadzu, model LC-8A, Kyoto, Japan) equipped with an Empower computer program (Waters, Mildford, MA, USA). A PL HFIP gel column (Polymer Lab) and a refractive index detector (Shimadzu RID-10A, Kyoto, Japan) were employed. The polymer was dissolved and eluted in 1,1,1,3,3,3-hexafluoroisopropanol containing CF_3_COONa (0.05 M) at a flow rate of 1 mL/min (injected volume 100 μL, sample concentration 2.0 mg/mL). The number and weight average molecular weights were calculated using polymethyl methacrylate standards.

Calorimetric data were obtained by differential scanning calorimetry with a TA Instruments Q100 series (TA instruments, New Castle, DE, USA) with *T*_zero_ technology and equipped with a refrigerated cooling system (RCS, TA instruments, New Castle, DE, USA). Experiments were conducted under a flow of dry nitrogen with a sample weight of approximately 5 mg and calibration was performed with indium. *T*_zero_ calibration required two experiments: the first was performed without samples while sapphire disks were used in the second. Thermal characterization was conducted following a protocol consisting of a heating run (3 °C/min), a cooling run (3 °C/min) after keeping the sample in the melt state for 5 min to wipe out the thermal history, and a subsequent heating run (3 °C/min). Non-isothermal crystallization studies were performed by cooling the previously molten samples (5 min at 125 °C) at rates varying from 30 to 1 °C/min. 

The spherulitic growth rate was determined by optical microscopy using a Zeiss Axioskop 40 Pol light (Zeiss, Oberkochen, Germany) polarizing microscope equipped with a Linkam temperature control system (Tadworth, UK) configured by a THMS 600 heating and freezing stage connected to a LNP 94 liquid nitrogen cooling system (Tadworth, UK). Spherulites were grown from homogeneous thin films prepared from the melt. Small sections of these films were pressed or smeared between two cover slides and inserted into the hot stage, giving rise to samples with thicknesses close to 10 µm in all cases. Samples were kept at approximately 125 °C for 5 min to eliminate sample history effects. The radius of growing spherulites was monitored during crystallization with micrographs taken with a Zeiss AxiosCam MRC5 digital camera (Zeiss, Oberkochen, Germany) at appropriate time intervals. A first-order red tint plate was employed to determine the sign of spherulite birefringence under crossed polarizers.

In vitro hydrolytic degradation assays were carried out at a physiological temperature of 37 °C using a pH 7.4 phosphate buffer (Sörensen medium: 19.268 g of Na_2_HPO_4_·12H_2_O and 1.796 g of KH_2_PO_4_ in 1 L of deionized water) and a pH 11 from the Universal buffer (citrate-phosphate-borate/HCl) solution, mixing 20 mL of a stock solution with 14.7 mL of 0.1 M HCl and distilled water up to a volume of 100 mL. The stock solution (1 L) contained 100 mL of citric acid and 100 mL of phosphoric acid solution, each of which was equivalent to 100 mL NaOH 1 M, 3.54 g of boric acid, and 343 mL of 1 M NaOH. Samples were kept under orbital shaking in bottles filled with 50 mL of the degradation medium and sodium azide (0.03 wt %) to prevent microbial growth for selected exposure times. The samples were then thoroughly rinsed with distilled water, dried to constant weight under vacuum and stored over P_4_O_10_ before analysis. Finally, weight retention and molecular weight were evaluated. 

Time resolved SAXS (small-angle X-ray scattering) experiments were conducted at the NCD beamline (BL11) of the Alba synchrotron radiation light facility (Cerdanyola del Vallès, Spain). The beam was monochromatized to a wavelength of 0.1 nm. Polymer samples were confined between Kapton films and then held on a Linkam THMS600 hot stage (Tadworth, UK) with temperature control within ±0.1 °C. SAXS profiles were acquired during heating and cooling runs in time frames of 20 s and rates of 10 °C/min. The SAXS patterns were calibrated with diffractions of a standard of a silver behenate sample. The diffraction profiles were normalized to the beam intensity and corrected considering the empty sample background. The correlation function and corresponding parameters were calculated with the CORFUNC program for Fiber Diffraction/Non-Crystalline Diffraction provided by Collaborative Computational Project 13 (CCP13) (Chester, UK).

## 3. Results and Discussion

### 3.1. Melting and Crystallization of Poly(p-dioxanone)

**Figure 1:**
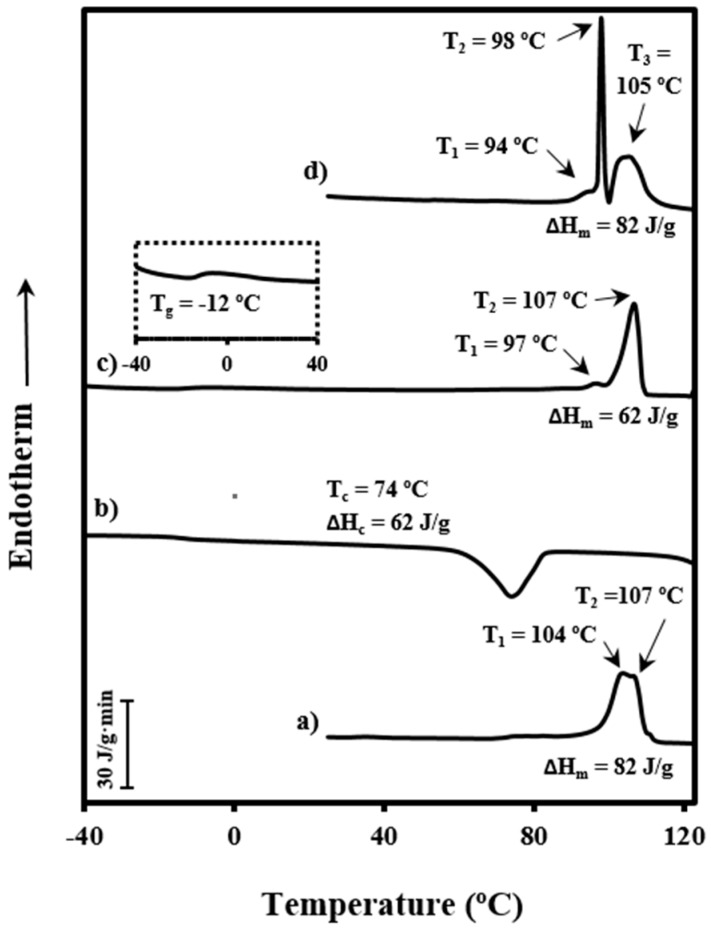
Differential scanning calorimetry (DSC) traces corresponding to heating runs of commercial granulated polydioxanone (PDO) (**a**) and PDO sutures (**d**), the cooling run of the melted granulated PDO (**b**) and the subsequent heating run (**c**). Glass transition can be detected in the magnification given in the inset in (**c**). All scans were performed at a rate of 3 °C/min.

Thermal behavior of PDO is rather complicated, as revealed by the DSC curves in Figure 1. Heating traces are clearly different for commercial granulated samples crystallized from the melt and processed surgical sutures, demonstrating the significant influence of thermal treatments on melting behavior. Thus, two well-differentiated endothermic melting peaks at 97 °C (small) and 107 °C were observed when samples crystallized from the melt state at a rate of 3 °C/min. These peaks have been largely discussed [6,7] and attributed to a typical lamellar reorganization process that leads to lamellar thickening during heating. Commercial granulated PDO showed that the high temperature peak was split into two equally intense peaks at 104 and 107 °C. Therefore, the population of thinner crystals was not present in the manufactured PDO form or at least they were sufficiently energetically unstable to lead to a complete recrystallization process on heating. Probably two populations of new crystals with practically the same thickness existed. The double peak appearing in the 104–107 °C range (see label at 105 °C) was also detected in the heating run of the as-processed suture, but in this case the population of thinner crystals was highly stable and did not undergo a recrystallization process. Note the intense and narrow peak at 98 °C, which in this case was observed as a consequence of the high temperature annealing process at which commercial sutures were submitted.

**Figure 2:**
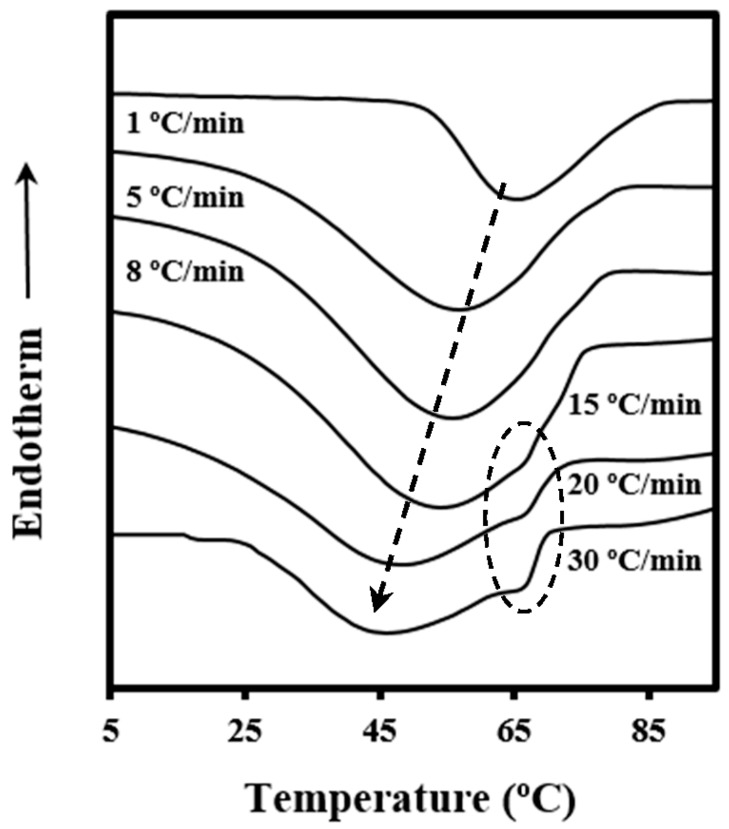
Exothermic DSC traces performed with PDO at the indicated cooling rates. The dashed ellipse contains the high temperature crystallization peak detected at high cooling rates, whereas the dashed arrow indicates the evolution of the main crystallization peak.

Figure 2 shows the dynamic DSC exotherms obtained by cooling the melted samples at different rates. Crystallization peaks progressively shift to lower temperatures as the cooling rate increases, as expected, but peaks become broader. Two different crystallization processes seemed to occur at cooling rates equal or higher than 15 °C/min, a feature that is later discussed on the basis of different nucleation mechanisms (i.e., instantaneous and sporadic at low and high temperatures, respectively).

### 3.2. Non-Isothermal Kinetic Analysis of Poly(p-dioxanone) Melt Crystallization from DSC Data

The process of crystallization under non-isothermal conditions is rather complicated to be analyzed since crystallization from the melt takes place under different degrees of supercooling, and therefore caution should be taken when interpreting experimental results.

Calorimetric data were used to determine the relative degree of crystallinity at any temperature, *χ*(*T*), for all cooling rates by the expression:

(1) $$\chi (T)=\frac{\int_{T_{0}}^{T_{c}}\left(dH_{c}/dT\right)dT}{\int_{T_{0}}^{T_{\infty }}(dH_{c}/dT)dT}$$

where d*H*_c_ is the enthalpy of crystallization released within an infinitesimal temperature range d*T*, *T*_0_ denotes the initial crystallization temperature and *T_c_* and *T*_∞_ are the crystallization temperatures at time *t* and after completion of the crystallization process, respectively. Thus, the denominator corresponds to the overall enthalpy of crystallization for specific heating/cooling conditions.

The relative degree of crystallinity was calculated as a function of time by the relationship

(*t* − *t*_0_) = (*T*_0_ − *T*)/*φ* (2)

where *T*_0_ is the temperature at which crystallization begins (*t* = *t*_0_) and *φ* is the value of the cooling rate. 

**Figure 3:**
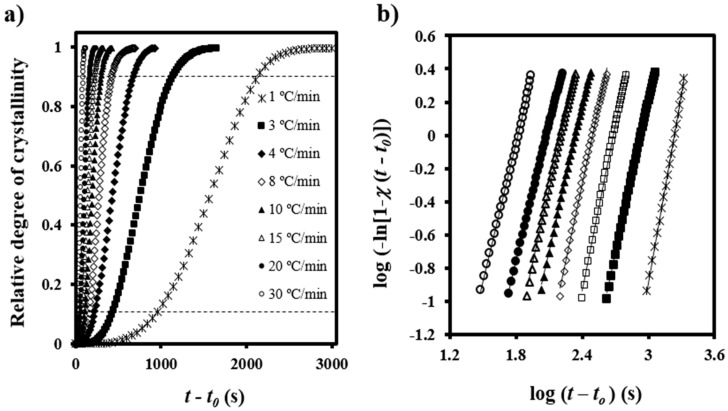
(**a**) Time evolution of relative crystallinity at the indicated cooling rates for non-isothermal crystallization of PDO; (**b**) Avrami analyses of non-isothermal crystallizations of PDO.

Figure 3a illustrates the variation of the time-dependent degree of crystallinity, *χ*(*t* − *t*_0_), at different cooling rates, which allows a typical Avrami analysis to be performed [20] according to the equation

1 − *χ*(*t* − *t*_0_) = exp[−*Z*(*t* − *t*_0_)*^n^*], (3)

where *Z* is the rate constant and *n* is the Avrami exponent. A normalized rate constant, *k* = *Z*^1/*n*^, is however more useful for comparison because its dimension (time^−1^) becomes independent of the Avrami exponent. 

Figure 3b shows the plots of log{−ln[1 − *χ*(*t* − *t*_0_)]} versus log(*t − t*_0_) at different cooling rates. A good linearity was observed between the relative degree of crystallinities of 0.10 and 0.90, that is, after formation of well-defined spherulitic morphologies and before occurrence of a secondary crystallization caused by the impingement of spherulites (see dashed lines in Figure 3a). 

Table 1 summarizes the main kinetic parameters calculated by the Avrami analysis. As known from isothermal studies, the normalized rate constant was low (i.e., between 0.58 × 10^−3^ s^−1^ and 15.26 × 10^−3^ s^−1^) and increased with the cooling rate. Avrami exponents showed a moderate variation (i.e., between 3.76 and 2.77), with the lowest values being determined for high cooling rates and the average value being close to 3.0. These exponents are lower than those previously reported by Zhang et al. [24] (i.e., 4.26–3.40) and in good agreement with those given by Andjelic et al. [4] for low crystallization rates (i.e., 3.0), although in this case a value of 1.1 was found for high crystallization rates. Isothermal crystallization studies also indicate slightly contradictory values. Thus, minimum changes with crystallization temperature were determined by Andjelic et al. (i.e., exponents varied between 2.22 and 2.62, with 2.5 being the average value) [4], but a systematic increase (i.e., from approximately 2 to 3.8) was also reported [6] for higher isothermal crystallization temperatures (i.e., from 30 to 80 °C). The last behavior was interpreted as a consequence of a change from instantaneous to sporadic nucleation as *T*_c_ was increased [6].

It can be well stated that application of the Avrami equation under non-isothermal conditions merely corresponds to a mathematical fitting that allows appropriate values of the rate constant to be derived [26,27,28]. In this case, it should be pointed out that the determined exponents may even have a physical meaning since they suggest three-dimensional spherulitic growth and instantaneous nucleation, as postulated from isothermal studies [4,6]. Furthermore, the sporadic nucleation detected at high isothermal crystallization temperatures [6] is in agreement with the increase of the exponent observed at low crystallization rates (i.e., 3.76 at 1 °C/min) and supports DSC evidence of the occurrence of two crystallization processes.

The values of the corresponding reciprocal crystallization half times (1/*τ*_1/2_), calculated as the inverse of the difference between crystallization starting time and crystallization half time, are also given in Table 1. This parameter is a direct measure of the crystallization process, and could therefore be used to check the accuracy of Avrami analyses, as demonstrated by the excellent agreement with the theoretical kinetic value (i.e., 1/*τ*_1/2_ = (*Z*/ln2)^1/*n*^). In conclusion, the deduced Avrami parameters are completely appropriate to simulate the non-isothermal crystallization process. 

A kinetic equation that combines the Avrami [20] and Ozawa [17] expressions has been derived and applied in different non-isothermal studies [29]:

log *φ* = log *F*(*T*) − *a* log(*t* − *t*_0_) (4)

where *F*(*T*) is a new kinetic parameter referring to the cooling rate which must be chosen at a unit crystallization time when the system reaches a certain crystallinity, and *a* is the ratio of apparent Avrami and Ozawa exponents.

**Figure 4:**
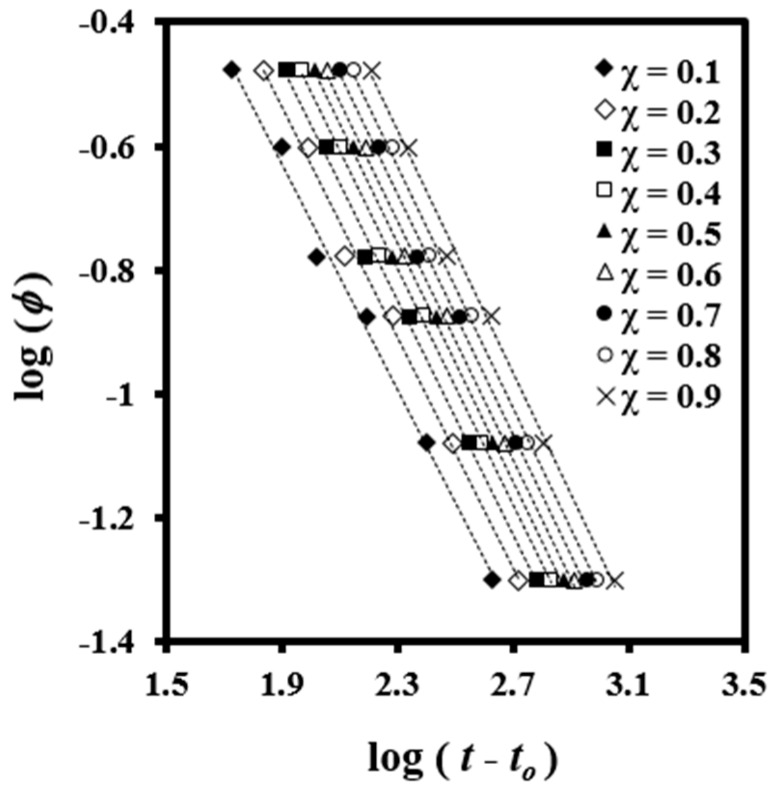
Plots of log (*φ*) versus log (*t* − *t*_0_) for non-isothermal crystallization of PDO performed at the indicated crystallinities.

A plot of log *φ* versus log (*t * − *t*_0_) yields a series of straight lines at a given value of *χ*(*T*) (Figure 4), which suggest the validity of the combined equation for this system. Kinetic parameters can be estimated by the intercept and slope of these lines. 

Results showed that *F*(*T*) values increased with crystallinity (Table 2), which has a physical sense because the motion of the molecular chains became slower as the material crystallized and formation of new crystals was hindered. The values of *a* were almost constant between 0.91 and 0.98 although slightly increased with the relative degree of crystallinity.

**Figure 5:**
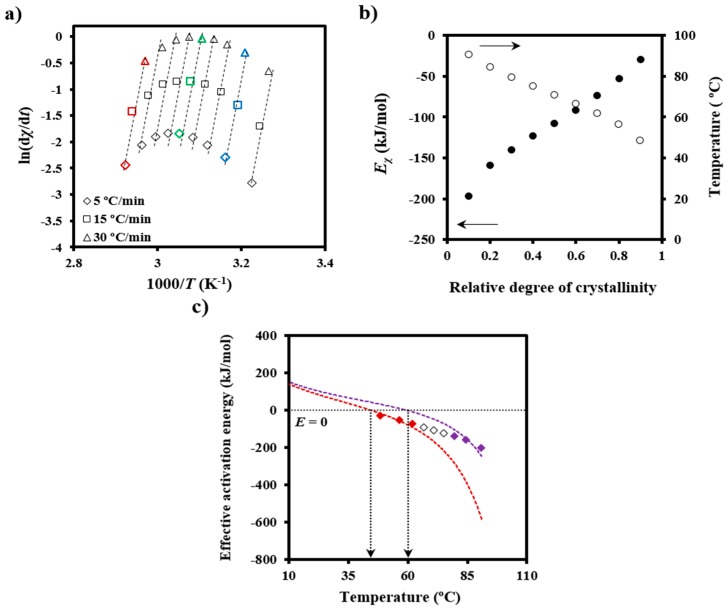
(**a**) Plots of ln[d*χ*/dt]*_χ_* versus 1/*T* for non-isothermal crystallization of PDO at the indicated cooling rates. Data corresponding to relative degrees of crystallinity of 0.8, 0.5 and 0.1 are represented by blue, green and red symbols, respectively; (**b**) Dependence of the activation energy of crystallization (●) and average temperature (○) on crystallinity; (**c**) Experimental *E_χ_* versus *T* plot and simulated curves according to Equation (6). Red and violet dashed lines indicate the simulated curves for regimes III and II, respectively. Arrows indicate the expected temperatures for the maximum crystallization rates (i.e., effective activation energy equal to zero).

The crystallization process has non-Arrhenius behavior, and therefore a temperature-dependent *effective activation energy* needs to be defined. The value corresponding to a given degree of crystallinity, *E_χ_*, can be determined by the Friedman isoconversional method [30]:

[d*χ*/d*t*]*_χ_* = *A*exp(−*E_χ_*/*RT*)*f*[*χ*], (5)

where *A* is a pre-exponential factor and *f*[*χ*] is the crystallization model. Values of ln[d*χ*/d*t*]*χ* at different temperatures and degrees of crystallization can be obtained from crystallization experiments performed at different cooling rates. In this way, the slopes of the linear plots of ln[d*χ*/d*t*]*_χ_* versus 1/*T* (Figure 5a) allow *E_χ_* to be determined (Figure 5b). The temperature dependence of the effective activation energy (Figure 5c) could finally be derived by considering also the average temperature associated with a given conversion (Figure 5b).

The effective activation energy was negative for crystallization experiments performed from the melt state and at low supercooling degrees (i.e., the temperature range where secondary nucleation plays a fundamental role), as shown in Figure 5c. This energy increased progressively (i.e., the crystallization rate increased) as the temperature decreased, as discussed at length by Vyazovkin and Dranca [31], reflecting the expected behavior for crystallizations performed at temperatures higher than those associated with the maximum crystallization rate. 

Vyazovkin and Sbirrazzuoli proposed that crystallization parameters like the secondary nucleation constant should be derived through the effective activation energies [31,32,33]:

*E*(*T*) = −*R*dln*G*/d*T*^−1^ = U*[*T*^2^/(*T* − *T_∞_*)^2^] + *K*_g_*R*[(2Δ*T* − *T*_m_°*f*)/(Δ*T*)^2^*f*] (6)

where *G* is the crystal growth rate, U* represents the activation energy characteristic of the transport of crystallizing segments across the liquid-crystal interface, *T*_∞_ is the temperature below which such motion ceases, *R* is the gas constant, *K*_g_ is the secondary nucleation constant, Δ*T* is the degree of supercooling measured as *T*_m_ − *T*_c_ (where *T*_m_ is the equilibrium melting temperature and *T*_c_ is the crystallization temperature), and *f* is a correction factor accounting for the variation in the bulk melting enthalpy per unit volume with temperature (*f* = 2*T*_c_/(*T*_m_ + *T*_c_)).

Figure 5c also compares the experimental *E_χ_* − *T* plot with simulated ones using Equation (6), U* and *T_∞_* values of 1600 cal/mol and *T*_g_-35 K, respectively (i.e., close to the universal values reported by Suzuki and Kovacs [34]), the equilibrium melting temperature of 127 °C, as previously determined for PDO [3], and representative *K*_g_ values. In fact, U* and *T_∞_* have little influence on a temperature range that is far from the glass transition temperature. The best fit between experimental and theoretical data was obtained considering two *K*_g_ parameters (i.e., 3.07 × 10^5^ K^2^ and 1.42 × 10^5^ K^2^), which are in agreement with the two crystallization regimes reported for PDO from isothermal crystallization experiments [3]. Note that the isoconversional analysis was able to detect the existence of several crystallization regimes and also to predict two maximum crystallization rates at temperatures of 45 and 60 °C, as deduced from the temperatures for each simulated curve where the effective activation energy was zero.

### 3.3. Non-Isothermal Kinetic Analysis of Poly(p-dioxanone) Melt Crystallization from Optical Microscopy Data

**Figure 6:**
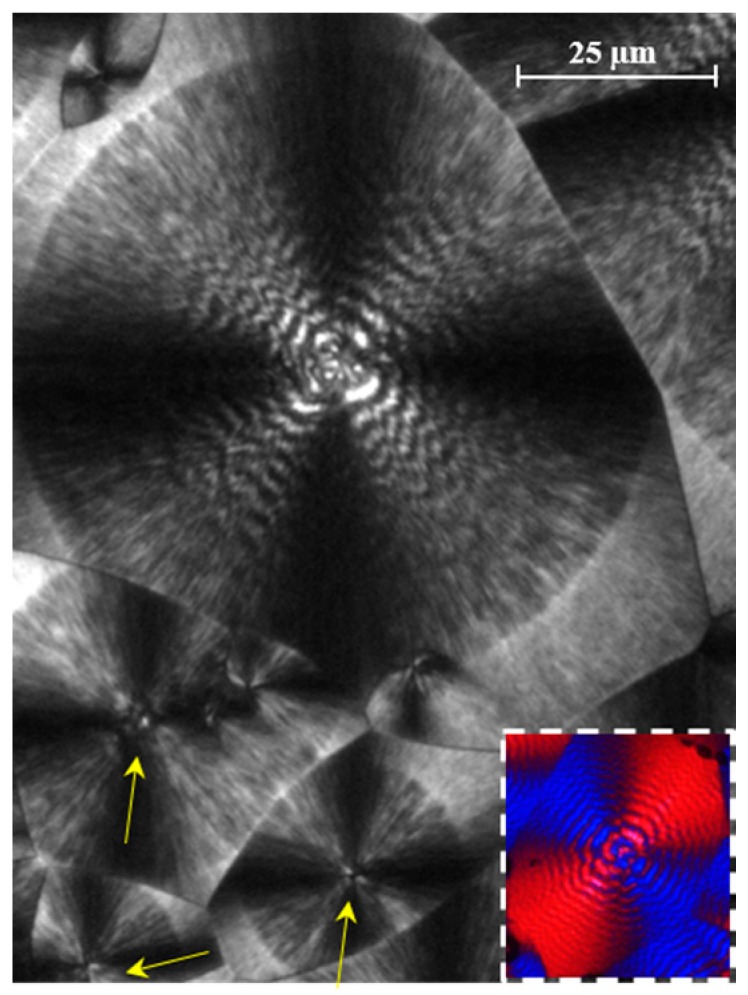
Optical micrograph of PDO spherulites formed during a non-isothermal crystallization from the melt state performed at a cooling rate of 20 °C/min. Yellow arrows point to spherulites formed at low temperatures. Inset shows a micrograph taken with a first-order red tint plate to determine the birefringence sign.

Non-isothermal crystallization of PDO rendered double banded spherulites with progressively decreasing periodicity (Figure 6), which is in accordance with the continuous temperature decrease of a non-isothermal crystallization. In fact, morphologies obtained under isothermal conditions have been extensively studied [3,5,6], and it was assumed that interband spacing decreased when crystallization temperature was lowered. Actually, two different banding periodicities could be detected where the broader bands had a negative birefringence. These kinds of double bands with uneven spacings are characteristic of spherulites having a biaxial indicatrix twisted about the optic normal [6,32,35]. PDO spherulites were also characterized by their big size, which led to poor nucleation and slow growth rate. Figure 6 also shows that the number of active nuclei increased during cooling, and consequently the size and morphology of the spherulites were not identical. Logically smaller spherulites with a practically indistinguishable double band texture were formed at lower temperatures (see yellow arrows). 

Spherulitic growth rates (*G*) were also determined for non-isothermal crystallization by measuring the change of the spherulite radius (*R*) with temperature (*T*) at a constant cooling/heating rate (d*T*/d*t*) [36,37]:

*G* = d*R*/d*t* = (d*R*/d*T*) × (d*T*/d*t*) (7)

**Figure 7:**
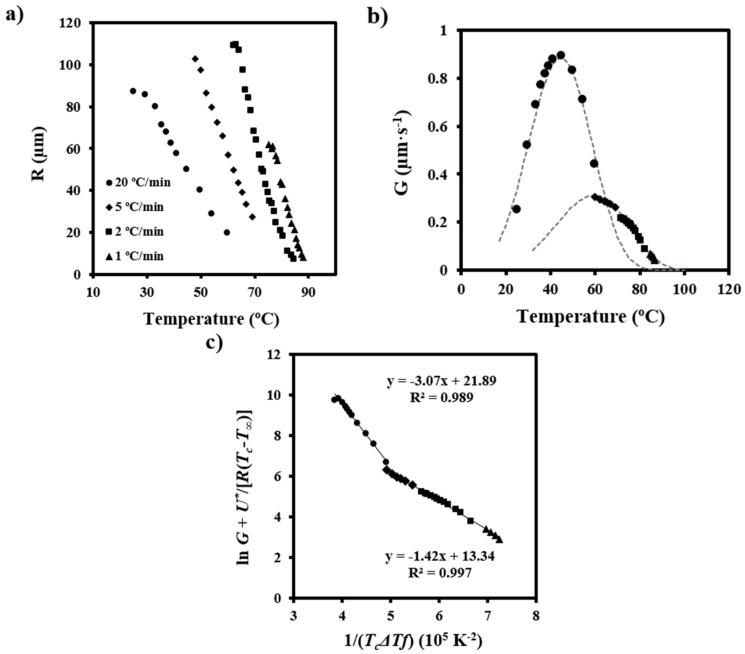
(**a**) Variation in spherulite radius with temperature during heating at the indicated rates; (**b**) Spherulitic growth rates determined by the equations deduced for the heating runs. Theoretical curves are also drawn (dashed lines) for comparative purposes; (**c**) Plot of ln*G* + U*/*R*(*T*_c_ − *T*_∞_) versus 1/*T*_c_(Δ*T*)*f* to determine the *K*_g_ secondary nucleation parameters of PDO.

Plots showing the variation of the spherulitic radius with crystallization temperature could be adjusted to third order equations with good regression coefficients (i.e., higher than 0.990) (Figure 7a). These coefficients were significantly better than those calculated for second order equations and remained constant for higher orders. Therefore, third order equations were employed to determine d*R*/d*T* as a function of the crystallization temperature. The corresponding crystal growth rate versus crystallization temperature curves are displayed in Figure 7b. Note that data were obtained at different cooling rates in order to maximize the crystallization temperature range where radii could be well measured.

Two bell-shaped curves with maximums of 45 and 60 °C reflected the temperature dependence of *G*, and therefore the existence of two crystallization regimes with different secondary nucleation constants. These were determined by the Lauritzen-Hoffman equation [38]:

*G* = *G*_0_exp[−U*/(*R*(*T_c_* − *T_∞_*))] × exp[−*K_g_*/(*T_c_*(Δ*T*)*f*)] (8)

where *G*_0_ is the constant pre-exponential factor and the other parameters as previously defined.

Figure 7c shows the linear plots obtained using U* and *T_∞_* parameters of 1600 cal/mol and *T*_g_-35 K, respectively. It is clear that two crystallization regimes defined by secondary nucleation constants of 3.07 × 10^5^ K^2^ and 1.42 × 10^5^ K^2^ fit the experimental data. Furthermore, regimes III and II could be assumed since the experimental ratio between slopes (2.16) was close to the theoretical *K*_g_^III^/*K*_g_^II^ value of 2. 

Figure 7b also shows that the two bell-shaped curves calculated by Equation (8), the estimated U* and *T*_∞_ parameters, and the deduced values of ln *G*_0_ and *K*_g_ for each regime fit well with the experimental spherulitic growth data. The maximum growth rate was found in regime III and corresponded to a temperature of 45 °C. Our observations are in agreement with the same crystallization regimes determined from isothermal crystallization although the nucleation constant becomes slightly higher than those previously reported (i.e., 2.49 × 10^5^ K^2^ and 1.19 × 10^5^ K^2^) [3]. Note also that the deduced data support the results of the calorimetric study.

The fact that the crystallization rate is governed by two different processes makes it unfeasible to determine a single activation energy for the entire *T*_c_ range. Instead, an effective activation energy (*E*) dependent on *T*_c_ was evaluated by Equation (9) [32]:

*E* = −*R*dln*G*/d*T*^−1^ = U*T^2^/(*T* − *T*_∞_)^2^ + *K*_g_*R*[(*T*_m_°)^2^ − *T*^2^−*T*_m_*T*]/[(*T*_m_ − *T*)^2^*T*] (9)

**Figure 8:**
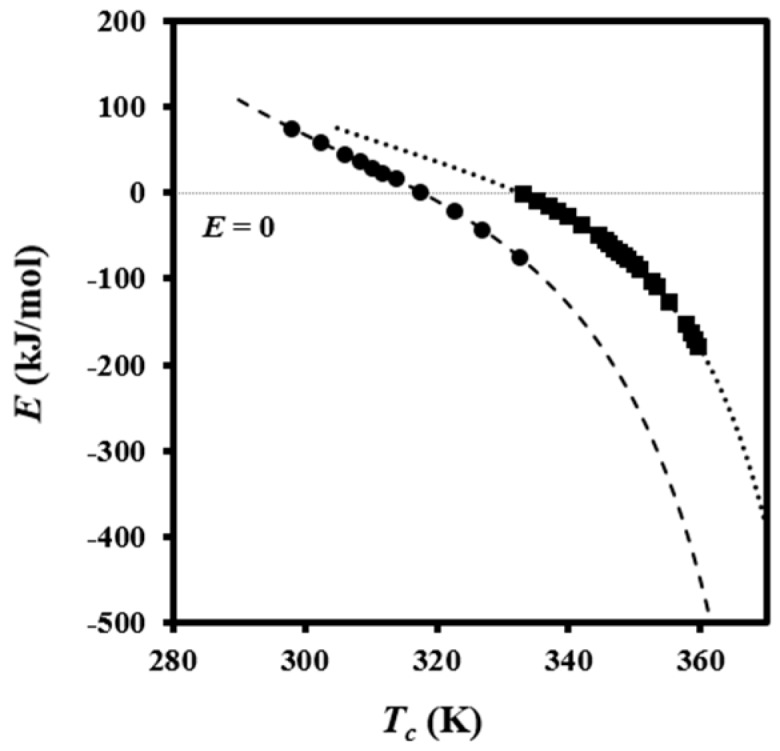
Dependence of effective activation energy on crystallization temperature for regimes II (^■^) and III (•). Extrapolated data for regimes II and III are indicated by dotted and dashed lines, respectively.

The calculated effective activation energies are plotted in Figure 8 and show non-Arrhenius behavior as expected. Different *K_g_* values were used according to the crystallization regime. The effective activation energy is zero at the maximum crystallization rate for regimes III and II, which corresponds to temperatures of 45 and 60 °C and agree again with those deduced by isoconversional analysis. In each case, positive values are found for temperatures lower than the corresponding maxima because the crystallization rate increases with increasing temperature, whereas negative values are determined for higher temperatures characterized by a decrease of the crystallization rate with increasing temperature. 

### 3.4. Evolution of Morphologic Parameters during Heating of Poly(p-dioxanone) Samples

**Figure 9:**
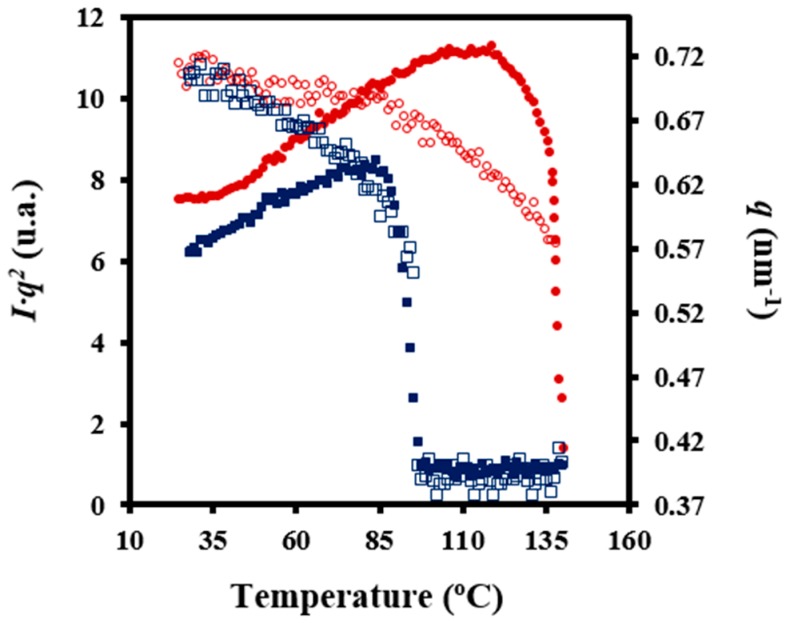
Variation of intensity (*Iq*^2^) (full symbols) and scattering vector (*q*) (empty symbols) of SAXS (small-angle X-ray scattering) peaks observed in the diffraction profiles taken during heating (10 °C/min) at room temperature (red) and during cooling (2 °C/min) from the melt state (blue).

Figure 9 shows the evolution of the intensity of the peak detected in SAXS patterns during heating and cooling processes of the granulated PDO sample. In the first case, a recrystallization process that led to thicker lamellae can be deduced from the increase in SAXS peak intensity and its shift towards lower values of the scattering vector (*q* = [4π/*λ*] senθ). In the second case, a shift of the peak once samples were crystallized towards higher *q* values and a slight decrease on its intensity was observed. These features can be explained considering that a lamellar insertion mechanism took place at low temperatures together with a densification of the amorphous phase (i.e., a smaller difference between the density of amorphous and crystalline phases).

Characteristic lamellar parameters (i.e., long period, *L_γ_*, amorphous layer thickness, *l_a_*, and crystalline lamellar thickness, *l_c_*) and crystallinity (i.e., crystallinity within the lamellar stacks, *X_c_^SAXS^* = *l_c_*/*L_γ_*) were determined by the normalized one-dimensional correlation function [35], *γ*(*r*):

(10) $$\gamma (r)=\int_{0}^{\infty }q^{2}I(q)\cos(qr)dq/\int_{0}^{\infty }q^{2}I(q)dq$$

where *I*(*q*) is the intensity at each value of the scattering vector.

SAXS data were collected within a limited angular range, with application of the Vonk’s model [39] and Porod’s law to perform extrapolations to low and high *q* values.

**Figure 10:**
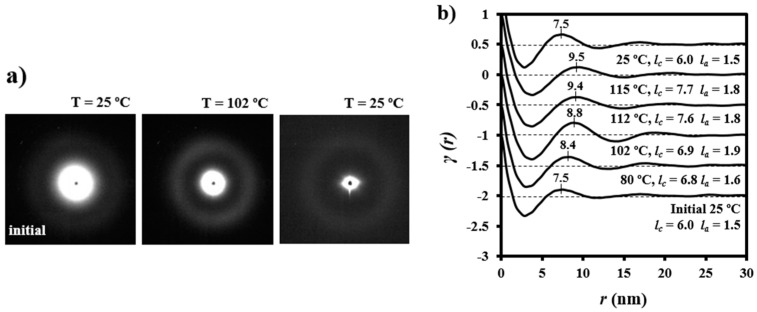
(**a**) SAXS patterns of a granulated PDO sample taken at 25 and 102 °C during a heating run performed at 10 °C/min; (**b**) Change in the correlation function during the heating run. For the sake of completeness the pattern and correlation function obtained at room temperature after cooling (10 °C/min) a previously molten sample are also shown.

Figure 10 illustrates representative correlation functions obtained from patterns acquired during the heating of granulated PDO. Lamellar thickening was due to the increase in crystalline lamellar thickness (i.e., from 6.0 to 7.7 nm) and amorphous layer thickness (i.e., from 1.5 to 1.8 nm). In this way, the reordering process led to a minimum increase of the crystallinity within lamellar stacks (i.e., from 0.80 to 0.81). However, it should be pointed out that the correlation function of the sample heated up to 102 °C (i.e., just before the first melting peak observed in the DSC trace) exhibited more defined peaks (see also the diffraction patterns in Figure 10) that were indicative of a high contrast between electronic densities of amorphous and crystalline phases. Basically, the amorphous phase became less dense in agreement with the maximum value detected for *l_a_* (i.e., 1.9 nm) and the minimum value of crystallinity (i.e., 0.78). Figure 10 also shows that morphological features were completely recovered after cooling the sample and specifically the correlation functions and the X-ray diffraction pattern were identical to those obtained from the initial sample. 

**Figure 11:**
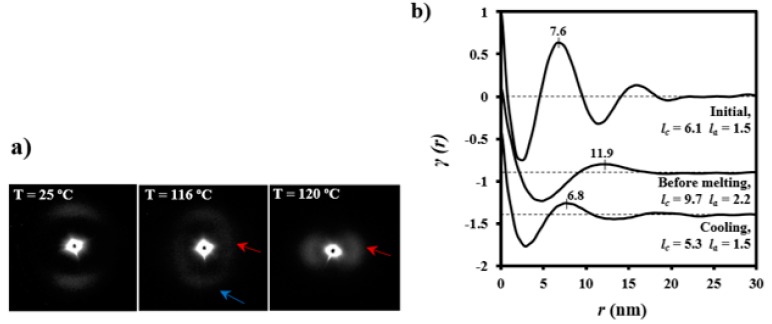
(**a**) SAXS patterns of a PDO suture taken at representative temperatures during a heating run at 10 °C/min. Blue and red arrows indicate meridional and equatorial reflections, respectively; (**b**) Correlation function of diffraction patterns corresponding to: the initial suture, a suture heated (10 °C/min) just before melting and a melt crystallized (cooling rate of 10 °C/min) suture at room temperature.

The evolution of SAXS patterns of a PDO thread on heating (Figure 11) was very different because they reflect the microstructure of the processed sample.

Some observations can be made: (a) The initial pattern was characterized by a meridional reflection that indicates a stacking of lamellae perpendicularly oriented to the fiber axis. Peaks observed in the corresponding correlation function were highly prominent suggesting a tie molecular arrangement in the dense crystalline phase. However, *l_c_* and *l_a_* values (6.1 nm and 1.5 nm) were close to those determined for the granulated sample; (b) As the temperature was increased the interlamellar reflection decreased in intensity while a new perpendicular reflection appeared and progressively increased in intensity. This new equatorial reflection could be associated with the existence of interfibrillar amorphous domains that correspond to the regions placed on the lateral sides of lamellae. Note that molecular chains in these domains may have had a partial orientation at the beginning of the heating (i.e., the as processed thread) but became more randomly distributed as the temperature was increased. Therefore, a decrease in electronic density of the amorphous phase and an enhancement of the intensity of the SAXS reflection were derived. The correlation function of the interfibrillar reflection observed in the pattern taken just before fusion gave *l_c_* and *l_a_* values (i.e., 9.7 nm and 2.2 nm, respectively), which were clearly different from those determined for the interlamellar reflection. In any case, *X_c_^SAXS^* was again close to 0.81; (c) The pattern and correlation function of the sample after being cooled to room temperature from the melt state were similar to those determined from the initial sample and indicated a similar lamellar organization. Nevertheless, a slight decrease of *l_c_* was detected (i.e., 5.3 nm as opposed to 6.1 nm) as well as a decrease of crystallinity within lamellar stacks (i.e., 0.78 as opposed to 0.80), in agreement with the lack of an annealing process for the melt crystallized sample. 

### 3.5. Evolution of Morphologic Parameters during Melt Crystallization of Poly(p-dioxanone) Samples

**Figure 12:**
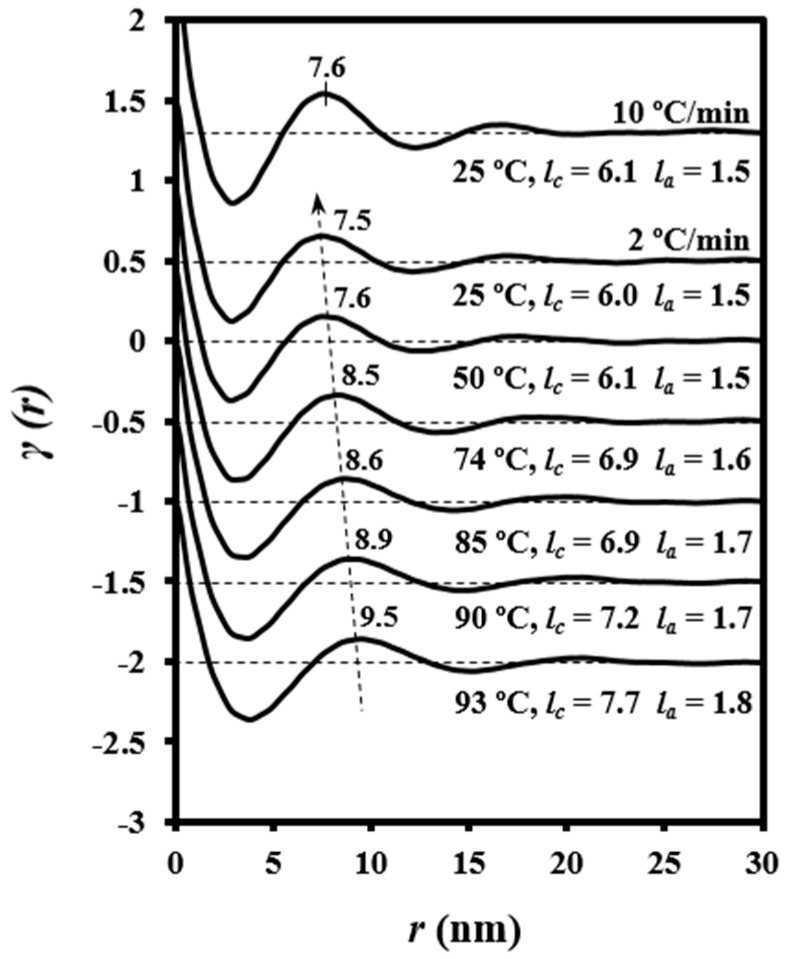
Correlation functions of patterns obtained at the indicated temperatures during the cooling run (2 °C/min) from the melt state. The correlation function of the pattern obtained at room temperature after cooling at 10 °C/min is also shown for comparative purposes.

Figure 12 illustrates the correlation functions from patterns taken during the cooling run (2 °C/min) of a melted PDO sample. It is clear that *L_γ_*, *l_a_*, and *l_c_* decreased progressively and had similar values (7.6, 1.5, and 6.1 nm) to those observed for the initial granulated PDO sample. The decrease in lamellar thickness can be due to the lower value expected when crystallization temperature decreases and also to a lamellar insertion mechanism (i.e., formation of thinner lamellar crystals between loosely stacked primary lamellae). Similar results were obtained at different cooling rates, but it is remarkable that morphological parameters were practically identical at room temperature, as shown in Figure 12 for the sample cooled at 10 °C/min. 

Note that crystallization took place at lower temperatures when the cooling rate was increased, and consequently lower lamellar thicknesses should be expected. Therefore, the invariance observed for *l_c_* suggests a counterbalance effect derived from the enhanced insertion mechanism (i.e., decrease of lamellar thickness) when the cooling rate was decreased.

### 3.6. Changes in Microstructure of Poly(p-dioxanone) Degraded Samples during Heating

**Figure 13:**
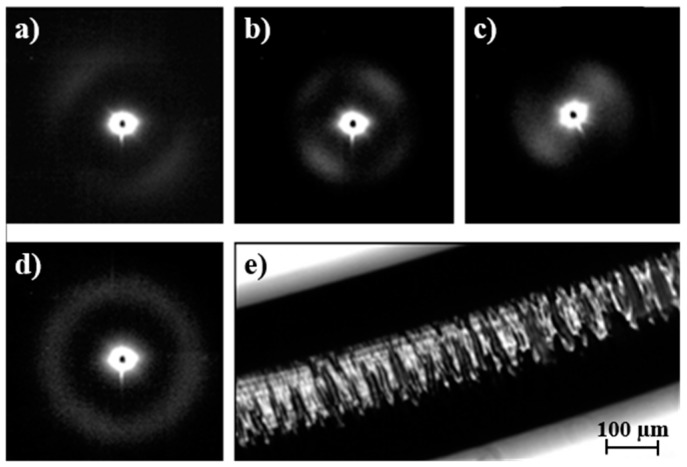
SAXS patterns taken at representative temperatures of 25 °C (**a**); 102 °C (**b**) and 107 °C (**c**) during a heating run at 10 °C/min of a PDO suture previously degraded in a pH 11 hydrolytic medium for 36 days. The pattern obtained at room temperature after cooling (10 °C/min) and the optical micrograph of the degraded suture are shown in (**d**,**e**), respectively.

**Figure 14:**
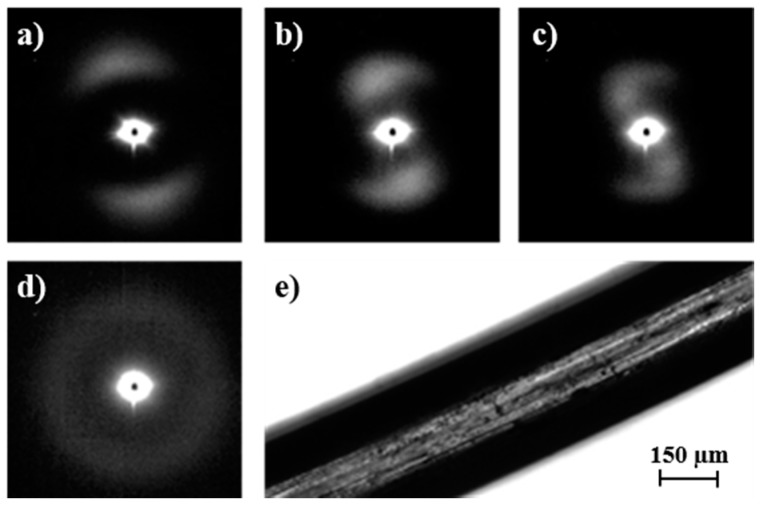
SAXS patterns taken at representative temperatures of 25 °C (**a**); 102 °C (**b**) and 112 °C (**c**) during a heating run at 10 °C/min of a PDO suture previously degraded in a pH 7 hydrolytic medium for 36 days. The pattern obtained at room temperature after cooling (10 °C/min) and the optical micrograph of the degraded suture are shown in (**d**,**e**), respectively.

Insights on the crystalline microstructure of sutures can be obtained following the evolution of SAXS patterns of degraded samples during a subsequent heating process [25]. To this end, PDO sutures were exposed to hydrolytic degradation media at pHs 7 and 11 and at 37 °C for 36 days to analyze samples with clear differences in their degree of hydrolysis. Specifically, *M*_w_ values of 117,700 and 83,800 g/mol were determined after exposure to pHs 7 and 11, respectively. Weight losses were close to 3% (pH 7) and 11% (pH 11). Micrographs in Figure 13 and Figure 14 reveal the greater morphological changes occurred under basic conditions and specifically the appearance of deep transversal cracks that led to narrow disks and tortuous suture surfaces (Figure 13). This degradation can be interpreted as a consequence of greater hydrolysis of interlamellar amorphous regions, which are depleted and dissolved in the medium [11]. The morphology of sutures exposed to neutral pH for a relatively short time is quite different because in this case smoother surfaces and numerous longitudinal cracks formed (Figure 14). In fact, it has been reported that hydrated, interfibrillar amorphous regions swell more easily than interlamellar amorphous regions [12]. Note that the latter are constituted by tie chains, which connect the lamellae in each fibril whereas fewer tie chains are expected to connect adjacent fibrils. Thus, water diffusion and formation of longitudinal cracks seem to be favored.

SAXS patterns of degraded samples showed a slight decrease of meridional interlamellar spacing, *L_B_*, because of the more compact structure achieved after scission of chains belonging to the amorphous regions. Therefore, the initial spacing of 9.6 nm decreased to 9.2 and 9.3 nm after 36 days of degradation in basic and neutral pH, respectively. Analysis of correlation functions (not shown) indicated that *l_c_* and *l_a_* values decreased from 6.1 to 1.5 nm, respectively, to 5.3 and 1.3 nm for the basic pH. Logically, SAXS crystallinity increased during degradation since the main change occurred in the amorphous layer.

Lamellar crystals in degraded samples were able to recrystallize and even reorient during subsequent heating runs in an easier way than that observed for the initial suture. The increased freedom resulting from scission of tie chains belonging to interfibrillar and interlamellar amorphous regions should play a fundamental role. Diffraction patterns during subsequent heating showed clear differences between highly and scarcely degraded samples. Thus, in the first case, an increase in lamellar thickness, together with the appearance of an intense equatorial reflection associated with the interfibrillar spacing previous to the disappearance of the meridional reflection, was observed. Specifically, *L_B_* values of 13.2 and 11.7 nm were determined at temperatures close to fusion (i.e., 102 °C). This behavior was similar to that observed for the initial suture taking into account the differences in spacings and intensities of reflections. Logically, greater spacings and intensities were detected for degraded samples as a consequence, in the first case, of an enhanced reorganization when tie interconnecting chains were cleaved and, in the second case, of a higher contrast between amorphous/crystalline regions.

Figure 14 reveals a different evolution when samples were degraded in the pH 7 medium because the great thickening of lamellae was hindered due to a still compact chain packing (note that weight loss was minimal). Therefore, the thickened lamellae (*L_B_* = 11.4 nm) tilted with respect to the fiber axis gave rise to a second reflection. The breadth of reorganized crystals was also clearly increased, as could be deduced from the observed spot like reflections. Finally, Figure 13 and Figure 14 show the reversibility of the thermal process for degraded samples since the diffraction patterns obtained after cooling to room temperature are identical to those obtained from the initial sample before any thermal treatment.

## 4. Conclusions

PDO showed complex melting and crystallization peaks because of a typical lamellar thickening process and the existence of different nucleation mechanisms, respectively. Calorimetric analysis of non-isothermal crystallization showed an increase of the Avrami exponent at low cooling rates, which could be associated with a homogeneous nucleation process instead of the instantaneous nucleation observed at high rates. Isoconversional analyses from non-isothermal calorimetric data revealed the existence of two crystallization regimes, demonstrating the suitability of this methodology. These regimes were well characterized by optical microscopy observations, and the non-isothermal crystallization results were in relatively good agreement with those previously reported from isothermal studies.

Real time SAXS profiles taken during heating and cooling processes showed the occurrence of a lamellar reordering process and a lamellar insertion mechanism that led to an increase and a decrease in lamellar thickness, respectively. SAXS patterns taken during heating of samples degraded under neutral and basic pH had a different evolution that revealed the existence of interlamellar and interfibrillar amorphous domains. 

## Figures and Tables

**Table 1:** Main non-isothermal crystallization kinetic parameters of polydioxanone (PDO) determined by differential scanning calorimetry (DSC).

φ (°C/min)	*n*	*Z* (s*^−n^*)	*k* × 10^3^ (s^−1^)	*τ*_1/2_ (s)	(1/*τ_1_*_/2_) × 10^3^ (s^−1^)	(*Z*/ln2)^1/*n*^ × 10^3^ (s^−1^)
1	3.76	6.79 × 10^−13^	0.58	1,574	0.64	0.64
3	3.11	7.90 × 10^−10^	1.18	749	1.34	1.32
5	3.30	1.37 × 10^−9^	2.06	428	2.34	2.31
8	3.10	1.83 × 10^−8^	3.21	272	3.68	3.61
10	2.92	1.45 × 10^−7^	4.55	192	5.21	5.16
15	2.97	2.76 × 10^−7^	6.17	142	7.07	6.98
20	2.73	2.25 × 10^−6^	8.60	104	9.65	9.83
30	2.77	9.12 × 10^−6^	15.26	58	17.14	17.42

**Table 2:** Values of kinetic parameters at a given crystallinity estimated from the combined model [35] for non-isothermal crystallization of PDO.

*χ*(*T*)	*a*	*F*(*T*)	*r*^2^
0.1	0.91	12.67	0.993
0.2	0.93	16.98	0.995
0.3	0.94	20.79	0.996
0.4	0.95	24.27	0.996
0.5	0.96	27.64	0.996
0.6	0.96	31.12	0.996
0.7	0.97	35.04	0.995
0.8	0.98	41.51	0.995
0.9	0.98	48.11	0.993
